# The untapped potential of inter-project cooperation of citizen science projects in Austria

**DOI:** 10.1177/09636625251410468

**Published:** 2026-01-26

**Authors:** Barbara Heinisch, Florian Heigl, Daniel Dörler

**Affiliations:** 1 University of Vienna, Austria; 2 BOKU University, Austria

**Keywords:** citizen science network, citizen science platform, cooperation, research cooperation

## Abstract

Despite the growing interest in citizen science, many projects continue to operate in isolation. This study explores the current state and potential for cooperation among citizen science projects in Austria by analyzing the extent, reasons and obstacles for cooperation. Through a questionnaire distributed to 121 projects listed on the Austrian citizen science platform *Österreich forscht*, 50 projects were examined. The analysis found that interactions between these projects are limited, with most cooperation focusing on sharing experiences. The primary motivation for (future) cooperation is achieving common goals, while the main obstacle to cooperating with other citizen science projects is a lack of capacity and resources. The role of the citizen science platform for increasing cooperation ranges from networking (events) to highlighting long-term projects that have the necessary infrastructure for cooperation. Future research could expand to projects outside the platform and examine the characteristics of collaborators.

## 1. Introduction

Citizen science has revolutionized the landscape of academic research by actively engaging members of the public in academic research (Heigl et al., 2019). As the number of citizen science projects and initiatives is increasing, the question arises whether some of these projects are reinventing the wheel because the potential for inter-project cooperation remains largely unexplored, particularly within national contexts. This paper delves into the untapped potential of cooperation among Austrian citizen science projects.

While there is no universal definition, cooperation generally refers to joint efforts by different actors who may pursue individual goals, focusing on assistance rather than co-creation (Laudel, 1999). Collaboration, on the other hand, involves working toward shared goals, characterized by collective ownership, resource pooling, consensus-building, and interdependence due to the division of labor (Dietrich et al., 2010; Katz and Martin, 1997; Laudel, 1999). In this study (which was conducted in German), the term "cooperation" is used broadly, reflecting the German term "Kooperation", which encompasses both cooperation and collaboration.

Collaboration success depends on several factors. Rybnicek and Königsgruber (2019) identify four key factors: institutional, relationship, output, and framework factors. These include institutional flexibility, trust, clear goal-setting, and a supportive environment. Similarly, Marek et al. (2015) propose a seven-factor model emphasizing context, membership, process and organization, communication, function, resources, and leadership. Both frameworks highlight the importance of trust, shared goals, and effective leadership in fostering collaboration.

Despite its benefits, cooperation in research projects faces challenges (vom Brocke and Lippe, 2015). These include managing uncertainty in methods and outcomes, balancing creative freedom with control, and addressing the diverse expectations of geographically dispersed partners. Process-related costs, such as time and emotional energy for coordination, and outcome-related costs, such as project delays or failures, further complicate collaboration (Dietrich et al., 2010; Fox and Faver, 1984). The literature (Rybnicek and Königsgruber, 2019) highlights that different forms of cooperation (whether academic, industry-based, etc.) exhibit numerous commonalities in terms of both benefits and challenges.

Collaboration is increasingly vital in academic research, enhancing innovation (Lin, 2017), economic development (Rossoni et al., 2023), and knowledge production. By pooling resources, sharing data, and leveraging diverse expertise, researchers can avoid duplication of effort and produce high-quality outcomes (Marek et al., 2015; Vaudano, 2020). These benefits are equally relevant for citizen science projects, which could collaborate on data, methods, infrastructure, and participants to maximize impact. Although academic research is an increasingly collaborative endeavor, the collaboration among researchers is an underexplored topic (Amundsen et al., 2019).

Although synergies between citizen science projects have been suggested (Heinisch and Seltmann, 2018a; Hummer and Niedermeyer, 2018), no national study has explored cooperation among such projects. This is particularly relevant as funding bodies often require collaboration between diverse stakeholders, including academia, civil society, and industry. Despite the widespread enthusiasm for citizen science, the extent and nature of inter-project cooperation remain underexplored, as projects often operate in silos, limiting the potential for inter-project cooperation.

As one of the first national citizen science platforms worldwide, *Österreich forscht* serves as an exemplary case study for analyzing cooperation between citizen science projects. The online platform *Österreich forscht*, launched in 2014, and the Citizen Science Network Austria (Dörler and Heigl, 2019) connect institutions from academia, research, education and practice, with three main objectives: (1) Strengthening citizen science in Austria; (2) Promoting quality in citizen science, and (3) Raising its profile nationally (Österreich forscht, 2024).

To achieve these objectives, cooperation is key, as outlined in the network's citizen science strategy. This strategy emphasizes mutual appreciation, openness, and the development of support measures to foster cross-project cooperation. Therefore, the findings of this study not only fill a research gap but also have both practical and strategic implications for *Österreich forscht*.

The study had two major aims: (1) to investigate the extent of and types of cooperation between citizen science projects listed on *Österreich forscht*; (2) Another aim, being practical in nature, was to explore potential support measures to facilitate cooperation between partner institutions of the Citizen Science Network Austria. These measures were derived from the reasons mentioned by the respondents for (not) cooperating and the ideas suggested by the respondents.

## 2. Method

To analyze which projects were cooperating, a LimeSurvey questionnaire was sent to all coordinators of 84 ongoing and 37 already completed citizen science projects listed on *Österreich forscht* on 14 February 2024 (a reminder was sent on 27 February 2024). The questionnaire comprised 16 items in total, of which eight were open and eight closed items, comprising seven item blocks (for full questionnaire, see Supplemental material):

- General information, including name of the project(s) and of the host institution
- Levels and forms of cooperation, including whether they have already cooperated with other projects, and the type of cooperation
- Reasons for cooperating (or not cooperating)
- Challenges when cooperating
- Barriers for cooperating
- Support and synergies
- Final comment

The questionnaire items were derived from literature on collaboration and cooperation in and beyond academia, including indicators for determining cooperation by Laudel (1999), the good practice for university–industry collaboration (Barnes et al., 2002), as well as the success factors for collaboration according to Marek, Brock and Savla, 2015.

The study focuses on higher-level analysis since the respondents were guaranteed anonymity. The results are placed in the context of the current literature on cooperation.

## 3. Results

To investigate the extent of and types of cooperation between citizen science projects listed on *Österreich forscht*, overall, 69 projects responded, which left 50 projects, including 12 cooperating projects, for analysis after applying the exclusion criteria (see Supplemental material). The number of projects and the number of responses do not match because one respondent may have completed the questionnaire for multiple projects.

### The projects

**Figure 1.**
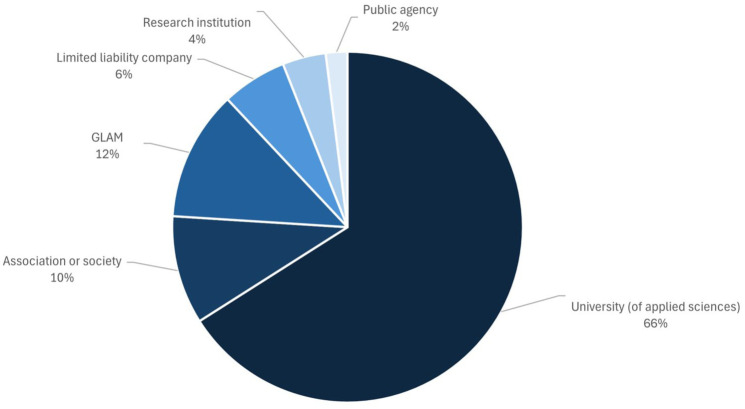
Types of institutions running citizen science projects that are listed on *Österreich forscht*.

The thematic range of the 50 projects analyzed reflects the general diversity of disciplines represented on the *Österreich forscht* platform, ranging from natural sciences, social sciences, and medicine to the humanities, among others. From an institutional perspective, the majority of the projects are run by a higher education institution (66%). Other organizations that run citizen science projects in Austria are associations or societies (10%) as well as galleries, libraries, archives, and museums (GLAM, 12%) (Figure 1). Moreover, the topics covered by the projects participating in the study are truly diverse, ranging from biodiversity and sociology to history and health. The projects differ in terms of project duration: from long-term projects, especially in the area of monitoring, to very recent initiatives.

Only about a quarter of all analyzed citizen science projects have experience in cooperating with other citizen science projects. Among the projects on *Österreich forscht* that are cooperating with others, the distribution of institutions is similar to that of the overall respondents. However, projects that are run by associations and societies stand out as they account for only 10% of the citizen science projects, and at the same time represent 34% of those projects that cooperate with other citizen science projects.

Regarding benefits, among the 12 projects on *Österreich forscht* that have already cooperated with others, half of them explicitly stated that the exchange (of methods, competences, data, or experience) was a key benefit of cooperating. Another benefit from cooperating was further development (either at the personal or the project level), such as gaining new experiences or insights, or benefiting from the expertise or data of the cooperation partners. Working toward a common (academic) objective or achieving greater impact (in society) were also mentioned as crucial benefits, as well as better funding opportunities or better geographical (data) coverage.

### Forms of cooperation

The survey revealed that among the 12 projects actively cooperating, ecology and biodiversity initiatives are the most likely to work together or share experiences. In this regard, projects with a broader (thematic) focus play a central role. For example, projects that cover a wide range of animal and plant species cooperate with those that study only individual species.

**Figure 2.**
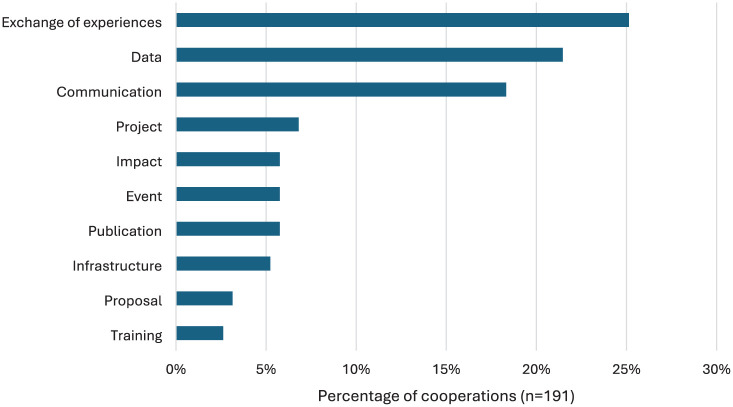
The forms of cooperation between citizen science projects.

Collectively, these 12 projects have shared experiences with 48 other projects on *Österreich forscht*, cooperated on data exchange or collection with 41 other projects, and implemented joint communication efforts with 35 projects. They have also undertaken joint projects with 13 other projects, co-published reports or academic papers, organized joint events, and achieved shared impact with 11 other projects each. They have shared infrastructure with 10 projects, submitted joint funding or project applications with six, and organized joint training sessions with five other projects (Figure 2).

### Reasons for cooperating

All 12 project coordinators who stated that they had already cooperated with other citizen science projects did so (Figure 4) because projects were pursuing a common goal. The second most common reason (eight responses) for cooperating with other projects on *Österreich forscht* was to use networks to reach more people or specific groups of people, followed by achieving societal impact (five responses) and a lack of know-how or expertise (three responses), for which the other project should compensate. Other reasons are that cooperation is required by funding bodies (two responses) or access to infrastructure, such as research data management, platforms, or technology through the other project (one response). None of the cooperating projects cooperated with others for the reason “lack of resources”, such as to compensate for a small budget or a lack of time, or personnel.

When being asked about the added value and benefits of previous cooperation, of the 12 projects that have already cooperated with others, some explicitly stated that exchanging methods, skills, data, or experience was beneficial. Furthermore, personal and project-level development plays a role, such as gaining new experience or insights, or benefiting from the expertise or data of the partners. Working toward a common academic goal or achieving greater impact in the public sphere through joint communication and advertising measures are just as important as better funding opportunities, as a joint project or better geographical (data coverage), both of which were mentioned several times.

While eight respondents reported no challenges in previous cooperation, four did: They struggled with a lack of communication or misunderstandings in communication (two responses) or pursued different objectives or had different priorities (two responses). Other obstacles experienced in previous cooperation were difficulties in coordinating and organizing the project. Moreover, different working methods and processes between the project partners led to difficulties in the project. The lack of support from superiors or support from the institution, as well as different levels of knowledge or expertise between the project partners (with one response each), were also mentioned as challenges when cooperating. However, one respondent explicitly stated that challenges can also be seen as an opportunity to learn and to make it better the next time.

### Reasons against cooperating

**Figure 3.**
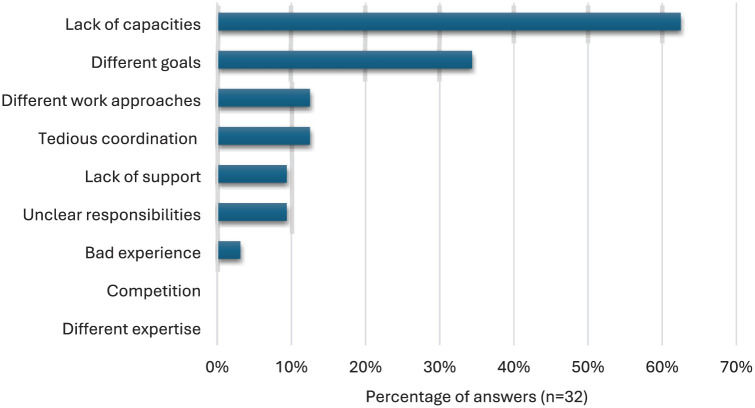
Reasons why the respondents do not cooperate with other citizen science projects.

Among the reasons speaking against cooperating (Figure 3), a lack of capacity and resources is the main reason (20 responses) for not entering into or initiating cooperation with other citizen science projects. The second most frequent reason against cooperation (11 responses) is different goals or priorities that are an obstacle to cooperating with other projects. In addition, the respondents gave the following reasons against cooperation: tedious coordination/management/organization (of the cooperation) and different working methods and processes (four responses each), unclear responsibilities and accountability, as well as lack of support from superiors or the institution (three responses each). One respondent would no longer cooperate due to bad experiences in the past. Different levels of knowledge/expertise or competitive thinking (e.g., competition for funding or theft of ideas) do not play a role.

Other reasons, mentioned in a separate questionnaire item, that speak against cooperation with other citizen science projects are a lack of thematic fit (four responses) or no necessity or no added value (two responses), as well as different ideas, lengthy preparatory work, changes of personnel among partners, unwillingness to cooperate, and the fact that working alone often allows for faster progress and means more independence.

### Why cooperate, nevertheless?

**Figure 4.**
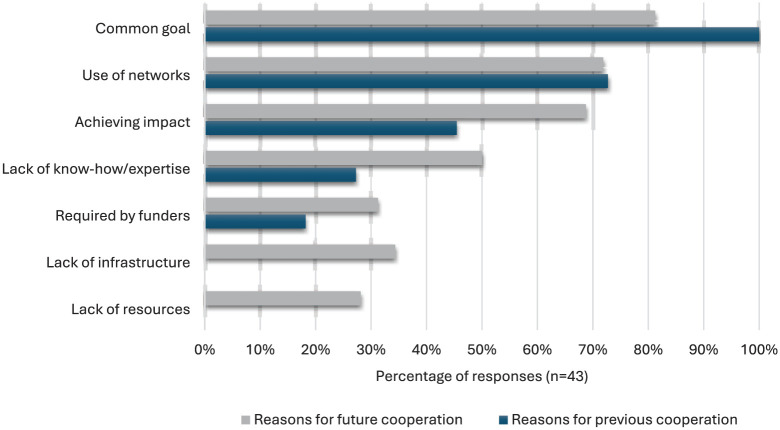
The respondents' reasons for previous cooperation and for potential future cooperation.

Among those projects that have not yet joined forces with other citizen science projects on *Österreich forscht*, some can certainly imagine doing so (Figure 4) if there is a common goal (26 responses) or they can make use of networks (e.g., to reach more people or a specific group of people) (23 responses). Achieving an impact (22 responses) and a lack of know-how/expertise (16 responses) that can be supplemented by the other partner might also lead to the initiation of cooperation.

When comparing the reasons for cooperation in the past and possibly in the future (Figure 4), we can see that a common goal, the use of networks, and the achievement of (societal) impact are at the top of the list in both cases.

The most frequently mentioned potential advantages of future cooperation relate to the fit between topic and objective. The projects hope to benefit from cooperation through the exchange of knowledge, experience, technology, and other resources. Increased visibility and outreach, the expansion of their networks, and reaching new target groups are also possible advantages identified by the respondents.

### How to strengthen cooperation on Österreich forscht

With regard to the second aim of the study, namely, to strengthen cooperation on *Österreich forscht*, the top priority for the respondents is networking. According to the respondents’ suggestions, this can take place, for example, at “platform meetings” (which are biannual meetings during which partners are informed about recent developments in the network and can discuss directions of future development for the network). With regard to networking, respondents also mentioned the creation of platforms where users can find cooperation partners or engage in exchange. This platform could list projects or people who are interested in cooperating with others. In a personal profile, users could highlight their previous experience, topics in which they are interested, and cooperation opportunities.

According to the respondents, it is particularly important to find projects that address a similar topic. Here, they suggested creating thematic working groups within the Citizen Science Network Austria, such as working groups specialized in, e.g., ecology, nature conservation, or medicine, or organizing workshops. Workshops could also be used to initiate cooperation, discuss successful case studies and best practices, or to showcase supply and demand in the field of citizen science (e.g., knowledge, expertise, technology, infrastructure), also to avoid duplication. Some respondents emphasized the importance of interdisciplinary networking and dedicated funding for cooperative projects.

## 4. Discussion

This study reveals limited cooperation among citizen science projects listed on *Österreich forscht*. When cooperation does occur, it often involves long-standing projects with overarching themes. Projects related to ecology or biodiversity that cover several animal or plant species stand out in particular. In the following, the findings are discussed in relation to the types of cooperation, added value, challenges, and strategies for strengthening cooperation across citizen science projects listed on *Österreich forscht*.

### Types of cooperation and motivations for (future) cooperation

The primary form of cooperation identified in this study was sharing of experiences, which can be referred to as “supportive cooperation” (Laudel, 1999), where the partner is not directly involved in executing the project but instead offers essential resources such as knowledge or infrastructure to support the research process of the partner. A subcategory, “service cooperation” (Laudel, 1999), involves providing access to resources (e.g., equipment or data) without requiring a shared research goal, which is also reflected in the study results.

Limited cooperation among projects listed on *Österreich forscht* may stem from the short-term nature of many citizen science initiatives, which often lack the resources and time to build sustained partnerships (unlike longer-term, better-funded projects).

The diversity of projects on *Österreich forscht* (spanning disciplines from natural sciences to the humanities) presents both opportunities and challenges for cooperation. While thematic alignment and shared goals were key drivers of cooperation, the study also found that some projects cooperated to achieve societal impact, such as influencing policy or raising public awareness.

#### Common goal

According to the results, one of the main reasons for previous cooperation or the initiation of future cooperation is a common goal that projects pursue. Although not explicitly stated by the respondents, the protection of biodiversity is presumably the unifying goal of cooperation for projects in the field of ecology. The interest in (societal) impact also falls into this category. This impact orientation leads to cross-institutional cooperation, e.g., between universities, authorities, associations, or museums. Many respondents who have not yet worked together with other citizen science projects on *Österreich forscht* could imagine cooperating with others if the topic and objective matched, and they experienced a certain added value.

However, the literature suggests that a common goal might not be a necessary precondition for cooperation. For example, one of the cooperating actors might follow a (research) aim while the other partner might only participate for and benefit from more favorable conditions provided through the cooperation (Laudel, 1999). However, the results of this study suggest otherwise, because all of the cooperating respondents cooperated with others because they followed a common goal or objective. This can be partly explained by the nature of the cooperation in this study: larger (biodiversity) projects encompassing several species might have the same goal as smaller (species-specific) projects, such as nature conservation or biodiversity protection. Also, pure research projects that want to understand the status quo (of a certain species) might do so in order to be able to inform policymakers and request and recommend protective measures, thus aligning with projects that are more oriented toward activism. To minimize the costs of cooperation, a shared goal should be clearly communicated from the outset in the form of a project vision, which “remains the most important success factor in all phases of the project” (vom Brocke and Lippe, 2015: 1030).

#### Resources

What emerges clearly from this study is that over a quarter of respondents from non-cooperating projects would engage with other citizen science projects in the future if they experience a lack of resources or infrastructure in their own project. However, the complete opposite applies to those projects that are already working together. Several respondents noted that cooperation requires additional resources, such as time or personnel for coordinative effort. Although cooperation can compensate for a lack of resources at one end, such as technology that a partner has, it requires extra resources at the other end for the purposes of coordination and organization. This might be the reason why some projects decide that the costs of cooperation are too high, and they prefer to work independently.

#### Previous experience

Regarding the (shared) history of cooperation, bad experience gained during previous cooperation was mentioned only once in this study, although Wu et al. (2024) suggest that this can be a major obstacle, as the benefits and costs of previous cooperation have a significant impact on the persistence of cooperation among scholars. In collaborative processes, people or institutions establish connections or build relationships (Dietrich et al., 2010).

The main reasons for cooperation in this study were a common goal, the use of networks and achieving impact as well as a lack of expertise or know-how. Therefore, the Citizen Science Network Austria may start to develop relevant measures, which may include information campaigns, a platform for exchange (of knowledge, experiences, and good practices), working groups (Heinisch and Seltmann, 2018b), further education and training, or (networking) events. These workshops or matchmaking events could help to identify and promote common goals among projects. Furthermore, Wood and Gray (1991) emphasize the role of the project leader since the role of the convener of a cooperation, as well as balancing individual self-interest with collective goals, is important for the success of (research) collaboration. The leader of a project is also responsible for ensuring a good division of labor (Fox and Faver, 1984). Therefore, the network might provide targeted training for (future) project leaders to ensure that this factor is covered.

In addition to citizen science networks, there are also other “interfaces” that facilitate cooperation between citizen science projects, such as shared staff and infrastructures, including application programming interfaces (APIs) as well as administrative units, e.g., at research institutions that “have an overview of projects [and] may see interfaces, overlaps and opportunities for collaboration” (Heinisch and Seltmann, 2018b: 42).

### Added value of cooperation

Cooperation offers numerous benefits, including resource sharing, knowledge exchange, and the potential for standardized methods. From the cooperation of different citizen science projects, several benefits and outcomes (of societal relevance) may emerge. However, only those will engage in collaboration who see a (long-term) benefit and added value (Barnes et al., 2002). To succeed, cooperation should incorporate factors that sustain partners’ interest and commitment.

#### (Mutual) benefits

Although different working methods and approaches were mentioned as a cooperation challenge in this study, the standardization of (data collection) methods (e.g., harmonization of protocols), for example, might allow for creating compatible data, allowing for direct comparison or combination of data over time and across different regions. For instance, the cross-referencing of data can enable cross-regional and temporal comparisons, as demonstrated by a successful cooperation between BOKU University, GeoSphere Austria, the Austrian Society for Nature Conservation, and the Natural History Museum Vienna, which has set itself the goal of better predicting the start of amphibian migrations. The analysis of more than 11,500 observations merged from different projects over 18 years showed that the flowering times of apricot trees and willows are good predictors of amphibian migration and can therefore provide the starting signal for protective measures (Peer et al., 2021).

Furthermore, training sessions, for example, in species identification, can be run jointly to allow participants to acquire the knowledge and skills necessary for participation (across projects). Highlighting and promoting these tangible benefits could encourage more projects to engage in cooperation. However, for collaboration to succeed, it must balance partners' goals and priorities while ensuring mutual benefits (Barnes et al., 2002).

#### Cooperation: A double-edged sword

This study revealed that each advantage a cooperation entails can also become a disadvantage. While cooperation can address resource gaps, it also imposes costs, such as the time and personnel required for coordination. While different backgrounds, including competences, cultures, and approaches, may enrich the cooperation, resulting in new ideas, findings, higher outreach, impact, and visibility or even capacity building (Heinisch and Seltmann, 2018a), they may, on the other hand, require more communication effort to clarify or avoid misunderstandings. Respondents noted that these demands often deter projects from cooperating, particularly when the perceived benefits do not outweigh the effort involved (Heinisch and Seltmann, 2018b).

#### Communication

Effective communication emerged both as a challenge and a key factor for successful cooperation in this study. Successful communication involves not only “speaking the same language” in terms of terminology (such as in interdisciplinary projects), but also managing the flow of information, choosing appropriate channels, and setting up regular meetings and reporting structures (Barnes et al., 2002).

Misunderstandings, diverging objectives (or priorities), and disciplinary jargon further complicate communication according to the study results, especially between academic institutions and non-governmental organizations (NGOs). Also, differing communication styles, such as public versus academic communication styles, can lead to complications in cooperation. While communication is challenging, “[m]ore frequent communication is associated with greater trust, respect, and participatory norms” (Cummings and Kiesler, 2007: 1622).

Therefore, citizen science networks can play a crucial role in bridging institutional and disciplinary gaps. They may facilitate information flow and exchange through regular meetings or digital platforms, to ensure exchanges of information beyond the disciplinary and organizational boundaries of the projects. They may create frameworks to address differences in communication styles between academic institutions, NGOs, and public stakeholders, fostering mutual understanding through workshops or cross-sector training.

### Barriers to cooperation

The reasons speaking against cooperation with other citizen science projects in this study (e.g., a lack of capacity and resources, different goals or priorities) were also identified previously (Heinisch and Seltmann, 2018b), where different motivations and diverging goals, backgrounds, project cultures, and terminology were considered drawbacks of cooperation. Also, increased administration and coordination for collaborative endeavors and competition (for participants and ideas) were mentioned in the previous study (Heinisch and Seltmann, 2018b).

The “costs” of collaboration increase when multiple organizations (especially very distinct ones, such as universities and NGOs) work together because both complexity and the difficulty of coordination increase, including the difficulty of integrating different expertise (Cummings and Kiesler, 2007). This perspective was also reflected in the responses of participants who questioned the added value of cooperation, noting the substantial preparatory effort involved and the relative efficiency of working independently. These views align with existing literature, which highlights the costs of collaboration in terms of coordination demands (Cummings and Kiesler, 2007), increased risk of project delays, potential failures (Guzzini and Iacobucci, 2016), or disagreements (Fallis, 2006) arising from interdependence. This raises the question of whether the projects yield greater benefits or more effective outcomes when operating independently or through cooperative engagement.

The coordination and management of the joint endeavor was considered a major obstacle by the respondents. Research coordination requires rules of cooperation and communication (Laudel, 1999). Successful collaboration requires establishing mutually agreed objectives based on common interests and strategic importance, supported by a well-developed project plan (Barnes et al., 2002). A factor jeopardizing coordination and management is institutional instability, that is, the partners are distracted by other issues within their organization, meaning they cannot pay the required attention and commitment to a cooperative effort. This is also emphasized by the fact that many of the managers of projects listed on *Österreich forscht* are also responsible for hands-on project work (and not only for coordination). Furthermore, organizations undergoing change are unlikely to be reliable partners in a collaboration (Barnes et al., 2002). This also translates to (academic) projects as they are, by nature, temporary, and involve (personnel) changes.

To facilitate coordination, project management, and communication, national citizen science networks may assign personnel to instigate cooperation among their projects. In addition to consultants, brokers (Heinisch and Seltmann, 2018b) can help connect diverse groups that are otherwise not linked. They can act as intermediaries bridging gaps between different teams, disciplines, or power levels within the network. Brokers may enhance cooperation by managing boundaries, securing institutional support, acquiring funding, and promoting inclusivity, especially for lower-power partners (Gray, 2008). In addition, citizen science networks or research departments (at universities) may hire personnel responsible for enabling cooperative projects by identifying and bringing together suitable projects.

### Strengthening cooperation

One of the aims of the citizen science strategy of the Citizen Science Network Austria is to strengthen cooperation among its projects. Basically, a combination of factors determines the success of a collaborative effort, which can be summarized as the preconditions, the process, and the outcomes of collaboration (Wood and Gray, 1991). While national citizen science networks can hardly influence the process and outcomes of a cooperation, they may be able to intervene on the level of preconditions.

#### Finding and selecting partners

The choice of partners is one of the universal success factors for collaboration (Barnes et al., 2002). Finding and selecting a cooperation partner was also considered important by this study’s respondents: a recurring topic was finding thematically similar projects. Therefore, a thematic fit between projects may be a precondition for entering into a cooperation at all. Thematically similar projects may benefit from (each other’s or jointly collected) data or the same group of participants (Heinisch and Seltmann, 2018b; Seltmann and Heinisch, 2018). Previous studies (Smith et al., 2021) demonstrate a curvilinear relationship between cooperation and topic similarity, where initial increases in similarity enhance cooperation, but too much similarity leads to increased competition and decreased synergy. Thus, cooperation is most likely at moderate levels of topic overlap, which also explains the predominance of larger (biodiversity) projects among the cooperating projects as they encompass a broader range of species, overlapping with the focus of smaller, specialized projects. This moderate topic overlap fosters synergy while avoiding competition (Smith et al., 2021).

While thematic alignment was considered important, the predominant driver for cooperation was the pursuit of “a common goal”, as mentioned above. To facilitate the identification of suitable partners, participants proposed the creation of a dedicated platform, a kind of partner-matching tool, which would streamline connections between projects with complementary goals. Nevertheless, when evaluating a potential new partner, the following criteria (Barnes et al., 2002) might be used: the strategic significance of the partner, complementary expertise and skills (which was also essential in this study), evidence of previous successful cooperation, and alignment of their objectives with the overall goals of the partnership.

To facilitate partner matching, national citizen science platforms can provide recommendations for the choice of partners. *Österreich forscht* could establish a “virtual marketplace” or partner search platform (Heinisch and Seltmann, 2018b). Long-term projects could be labeled as potential cooperation hubs, encouraging knowledge sharing and resource pooling. In addition, targeted training for project leaders could enhance their ability to balance individual and collective goals, ensuring effective coordination and division of labor.

#### Preconditions for cooperation

Apart from the factors mentioned above, there are also factors that cannot be easily influenced, which Rybnicek and Königsgruber (2019) termed “framework factors”. These include geographical and institutional aspects that cannot “be easily alleviated by providing more shared resources or forcing communication” (Cummings and Kiesler, 2007: 1632). Although *Österreich forscht* can hardly intervene on these framework conditions, despite lobbying and outreach, it still can continue informing its members about current developments because for successful cooperation, it is essential that partners stay informed about current developments, as these can significantly impact outcomes and present strategic opportunities (such as funding or policy changes) that should be actively monitored and leveraged (Rybnicek and Königsgruber, 2019). Thus, staying responsive to these external factors is essential for successful cooperation.

### Limitations and future research

This study is limited in its scope as it focuses exclusively on projects listed on *Österreich forscht*, excluding the broader spectrum of citizen science projects across Austria. In addition, the study did not consider the collaboration beyond the Citizen Science Network Austria, such as with research projects that do not have a citizen science element, as well as with citizen science projects outside of Austria (which was also highlighted by one respondent). An interesting observation in this study was that some projects claimed they did not cooperate with other citizen science initiatives, while others reported cooperation with the same projects. A possible reason for this discrepancy is that respondents might not have been aware of ongoing cooperation schemes, possibly due to staff changes within the projects.

Future research could broaden the scope to include European cooperation endeavors, explore demographic factors and career stages of collaborators (Subramanyam, 1983; Wu et al., 2024), for example, whether junior scholars are more likely to engage in collaborative research endeavors compared to senior scholars (Viale, 2010), and assess how these elements, alongside topic proximity, influence cooperation. Interpersonal trust, while not a direct focus of this study, emerged as a key element for successful cooperation. Building trust requires time and continuity, which short-term projects may struggle to provide. Future studies could investigate how trust develops in citizen science cooperation and its impact on long-term partnerships.

## 5. Conclusion

As citizen science projects expand in scope, the potential for inter-project cooperation becomes increasingly relevant, not only to enhance academic and societal outcomes but also to promote efficiency and shared learning. While cooperation can generate important benefits such as resource pooling, improved visibility, and innovation through diverse perspectives, it is not without significant costs. These include time-intensive coordination, the need for mutual adjustment of approaches and methods, and the risk of slowed progress due to divergent expectations, organizational cultures, or uneven levels of commitment. The findings indicate that, despite broad acknowledgment of its value, cooperation between citizen science projects in Austria remains limited, partly due to these institutional, relationship, output, and framework factors.

To move beyond fragmented efforts, future initiatives (such as those driven by the Citizen Science Network Austria) must weigh the costs of cooperation against its intended benefits and invest in frameworks that support long-term trust-building, capacity building, and provision of resources. Only by acknowledging and actively managing these tensions can the full potential of cross-project cooperation in citizen science be realized.

## Supplemental Material

sj-pdf-2-pus-10.1177_09636625251410468 – Supplemental material for The untapped potential of inter-project cooperation of citizen science projects in Austria Supplemental material, sj-pdf-2-pus-10.1177_09636625251410468 for The untapped potential of inter-project cooperation of citizen science projects in Austria by Barbara Heinisch, Florian Heigl and Daniel Dörler in Public Understanding of Science

sj-xlsx-1-pus-10.1177_09636625251410468 – Supplemental material for The untapped potential of inter-project cooperation of citizen science projects in Austria Supplemental material, sj-xlsx-1-pus-10.1177_09636625251410468 for The untapped potential of inter-project cooperation of citizen science projects in Austria by Barbara Heinisch, Florian Heigl and Daniel Dörler in Public Understanding of Science
