# Supplementary material for: The untapped potential of inter-project cooperation of citizen science projects in Austria
Source: Public Underst Sci. 2026 Jan 26;35(4):455–69. doi: 10.1177/09636625251410468 (PMC13096625; doi:10.1177/09636625251410468)
Supplement: sj-pdf-2-pus-10.1177_09636625251410468 – Supplemental material for The untapped potential of inter-project cooperation of citizen science projects in Austria [file sj-pdf-2-pus-10.1177_09636625251410468.pdf]

The Untapped Potential of Inter-Project Cooperation of Citizen Science Projects in Austria

Barbara Heinisch
Centre for Translation Studies, University of Vienna, Austria and
Institute of Zoology, BOKU University, Austria

Florian Heigl
Institute of Zoology, BOKU University, Austria

Daniel Dörler
Institute of Zoology, BOKU University, Austria

Corresponding author:
Barbara Heinisch, barbara.heinisch@univie.ac.at

Supplemental Material

This Supplemental Material for PUS-25-0022.R1 consists of:

1. Selection of questionnaire items 3

2. Questionnaire (in German) and its English translation (for information purposes only) 5

 2.1. Questionnaire overview in German 5

 2.2. Questionnaire overview in English (for information purposes only) 7

 2.3. Full questionnaire in German 9

  Kooperationen im Citizen Science-Netzwerk 9

  Allgemeine Angaben 9

  Ebenen und Formen der Kooperation bisher 9

  Gründe für Kooperation 10

  Herausforderungen bei Kooperation 11

  Barrieren für Kooperation 12

  Unterstützung und Synergien 12

  Abschluss 13

 2.4. Full questionnaire in English (for information purposes only) 14

  Cooperation in the Citizen Science network 14

  General information 15

  Levels and forms of cooperation to date 15

  Reasons for cooperation 16

  Challenges of cooperation 16

  Barriers to cooperation 17

  Support and synergies 18

  End of questionnaire 18

3. Inclusion and exclusion criteria 19

1. Selection of questionnaire items

Derived from the literature (see Methods), the following types of cooperation between (citizen science) projects have been identified (originally in German):

- Project = realization of a joint project
- Application = Submission of a (joint) funding proposal or project application
- Training = Offering joint training courses or programs
- Publication = Joint (academic) publication, such as academic articles, reports, recommendations, etc.
- Data = joint collection, use or exchange of data
- Communication = Recruitment of citizen scientists or project communication or public relations work or joint social media channels
- Events = realization of (joint) events
- Infrastructure = utilization or development or purchase of infrastructure and/or technology
- Consultancy = exchange of experience or recommendations
- Impact = achieving (societal) effect or impact

Reasons for cooperation:

- Lack of resources (too little money, time or personnel)
- Lack of infrastructure (e.g. research data management, platforms, technology)
- Common goal
- Required by funding bodies
- Lack of know-how/expertise
- Achieving (societal) impact
- Utilization of networks (e.g. reaching more people or a specific group of people)

Challenges of cooperation:

- Lack of or misleading communication with each other
- Coordination/management/organization
- Different working methods and processes
- Unclear competences and responsibilities
- Lack of support from superiors or the institution
- Different goals or priorities
- Different levels of knowledge/expertise

Barriers to cooperation:

- Lack of capacities/resources
- Difficult coordination/management/organization
- Different working methods and processes
- Unclear competences and responsibilities

- Lack of support from superiors or the institution
- Different goals or priorities
- Different levels of knowledge/expertise
- Competitive thinking (e.g. funding, theft of ideas)
- Bad experiences with previous collaborations

2. Questionnaire (in German) and its English translation (for information purposes only)

2.1. Questionnaire overview in German

Code	Frage	Fragetyp	Gruppe	Zwingend notwendig
KO01	Wie ist der Name Ihres aktuellen bzw. abgeschlossenen Citizen Science-Projekts?<br><br>An welcher Organisation ist bzw. war das jeweilige Citizen Science-Projekt angesiedelt?	Array (Texts)	Allgemeine Angaben	
KO03	Haben Sie bereits mit anderen Citizen Science-Projekten im Citizen Science Network Austria kooperiert?<br><br>(Unter „Kooperation“ verstehen wir die Zusammenarbeit zu einem bestimmten Thema oder in einem Projekt, die Organisation von gemeinsamen Veranstaltungen usw.)	List (Radio)	Ebenen und Formen der Kooperation bisher	ja
KO04MATRIX	Bitte wählen Sie alle Citizen Science-Projekte aus, mit denen Sie auf den folgenden Ebenen bereits kooperiert haben.<br><br>Projekt = Durchführung eines gemeinsamen Projekts<br>Antrag = Einreichung eines (gemeinsamen) Förder- oder Projektantrags<br>Schulungen = Anbieten von gemeinsamen Schulungen oder Trainings<br>Publikation = Gemeinsame (wissenschaftliche) Publikation, wie wissenschaftliche Artikel, Berichte, Empfehlungen usw.<br>Daten = gemeinsame Sammlung bzw. Nutzung bzw. Austausch von Daten<br>Kommunikation = Anwerbung von Citizen Scientists bzw. Projektkommunikation bzw. Öffentlichkeitsarbeit bzw. gemeinsame Social Media-Kanäle<br>Veranstaltungen = Durchführung von (gemeinsamen) Veranstaltungen<br>Infrastruktur = Nutzung bzw.	Array (Numbers)	Ebenen und Formen der Kooperation bisher	

	Entwicklung bzw. Ankauf von Infrastruktur/Technologie<br>Beratung = Erfahrungsaustausch bzw. Empfehlungen<br>Wirkung = Erzielung (gesellschaftlicher) Wirkung bzw. Impact			
KO07	Wie genau haben Sie kooperiert?	Long free text	Ebenen und Formen der Kooperation bisher	
KO08A	Warum haben Sie mit anderen kooperiert?	Multiple choice	Gründe für Kooperation	ja
KO08B	Warum würden Sie mit anderen kooperieren?	Multiple choice	Gründe für Kooperation	ja
KO09A	Welchen Nutzen hatten bzw. haben Sie von der Kooperation?	Long free text	Gründe für Kooperation	
KO09B	Welche Vorteile könnte Ihnen eine Kooperation bringen? Mit wem würden Sie kooperieren?	Long free text	Gründe für Kooperation	
KO10	Gab es Herausforderungen bei der Kooperation?	List (Radio)	Herausforderungen bei Kooperation	ja
KO11	Was waren die Herausforderungen bei der Kooperation?	Multiple choice	Herausforderungen bei Kooperation	ja
KO12	Das möchte ich zu den Herausforderungen der Kooperation noch ergänzen...	Long free text	Herausforderungen bei Kooperation	
KO13A	Warum haben Sie bisher nicht kooperiert?	Multiple choice	Barrieren für Kooperation	ja
KO14	Wie könnten Kooperationen im Citizen Science-Netzwerk verstärkt werden?	Long free text	Unterstützung und Synergien	Sanft
KO16	Ich kann Folgendes in das Citizen Science-Netzwerk einbringen...	Long free text	Unterstützung und Synergien	Sanft
KO17	Welche neuen Partner sollten als Institutionen ins Citizen Science Network Austria aufgenommen werden?	Short free text	Unterstützung und Synergien	
KO18	Das möchte ich noch ergänzen...	Long free text	Abschluss	

2.2. Questionnaire overview in English (for information purposes only)

Code	Question	Question type	Group	Mandatory
KO01	What is the name of your ongoing or completed citizen science project?<br>At which organization is, or was, the citizen science project implemented?	Array (Texts)	General information	
KO03	Have you already cooperated with other citizen science projects in the Citizen Science Network Austria?<br>(By "cooperation" we mean collaboration on a specific topic or in a project, the organization of joint events, etc.)	List (Radio)	Levels and forms of cooperation to date	yes
KO04MATRIX	Please select all citizen science projects with which you have already cooperated at the following levels.<br>• Project = implementation of a joint project<br>• Application = submission of a (joint) funding or project application<br>• Training = offering joint training courses or training sessions<br>• Publication = Joint (scientific) publication, such as scientific articles, reports, recommendations, etc.<br>• Data = joint collection, use or exchange of data<br>• Communication = recruitment of citizen scientists or project communication or public relations work or joint social media channels<br>• Events = organization of (joint) events<br>• Infrastructure = use or development or purchase of infrastructure/technology<br>• Advice = exchange of experience or recommendations<br>• Impact = achieving (social) effect or impact	Array (Numbers)	Levels and forms of cooperation to date	
KO07	Please specify how you cooperated in detail?	Long free text	Levels and forms of cooperation to date	
KO08A	Why did you cooperate with others?	Multiple choice	Reasons for cooperation	yes
KO08B	Why would you cooperate with others?	Multiple choice	Reasons for cooperation	yes
KO09A	How have you benefited from the cooperation?	Long free text	Reasons for cooperation	

KO09B	What benefits do you see in a future cooperation? Who would you cooperate with?	Long free text	Reasons for cooperation	
KO10	Were there any challenges in the cooperation?	List (Radio)	Challenges of cooperation	yes
KO11	What were the challenges of the cooperation?	Multiple choice	Challenges of cooperation	yes
KO12	I would like to add this comment on the challenges of cooperation...	Long free text	Challenges of cooperation	
KO13A	Why have you not cooperated so far?	Multiple choice	Barriers to cooperation	yes
KO14	How could cooperation within the Citizen Science Network be strengthened?	Long free text	Support and synergies	slightly
KO16	I can contribute the following to the Citizen Science Network...	Long free text	Support and synergies	slightly
KO17	Which new partners should be new institutions in the Citizen Science Network Austria?	Short free text	Support and synergies	
KO18	If you would like to add anything,...	Long free text	End of questionnaire	

2.3. Full questionnaire in German

Kooperationen im Citizen Science-Netzwerk

Als Citizen Science-Community sind wir an der ständigen Weiterentwicklung des Citizen Science Netzwerks Österreichs interessiert. Ein wesentlicher Bestandteil des Netzwerks ist die Kooperation untereinander und die Synergien, die zwischen Initiativen und Projekten entstehen können. Mit dieser Umfrage möchten wir daher den aktuellen Stand der Kooperationen und Synergien der Initiativen und Projekte untereinander erheben, um weitere Unterstützungsmöglichkeiten entwickeln und anbieten zu können.

Das Ausfüllen der Umfrage dauert **ca. 10 Minuten**.
Wir freuen uns, auf eure Antworten **bis 29. Februar 2024**.

Die Ergebnisse der Umfrage werden als Blogbeitrag auf Österreich forscht veröffentlicht und eine wissenschaftliche Publikation ist geplant. In beiden Fällen sind keinerlei Rückschlüsse auf individuelle Projekte möglich. Es werden lediglich allgemeine Aussagen gemacht, wie beispielsweise „Naturwissenschaftliche Projekte auf Österreich forscht kooperieren vor allem im Bereich XY".

Bei Rückfragen wenden Sie sich bitte an: [Name und E-Mail-Adresse der Wissenschafter*in]

Das Team von Österreich forscht
[Name der Wissenschafter*innen]

Allgemeine Angaben

Wie ist der Name Ihres aktuellen bzw. abgeschlossenen Citizen Science-Projekts?*

An welcher Organisation ist bzw. war das jeweilige Citizen Science-Projekt angesiedelt?*

Ebenen und Formen der Kooperation bisher

Haben Sie bereits mit anderen Citizen Science-Projekten im Citizen Science Network Austria kooperiert?*

(Unter „Kooperation" verstehen wir die Zusammenarbeit zu einem bestimmten Thema oder in einem Projekt, die Organisation von gemeinsamen Veranstaltungen usw.)

Bitte wählen Sie eine der folgenden Antworten:

- Ja
- Nein

Bitte wählen Sie alle Citizen Science-Projekte aus, mit denen Sie auf den folgenden Ebenen bereits kooperiert haben.*

- Projekt = Durchführung eines gemeinsamen Projekts
- Antrag = Einreichung eines (gemeinsamen) Förder- oder Projektantrags
- Schulungen = Anbieten von gemeinsamen Schulungen oder Trainings
- Publikation = Gemeinsame (wissenschaftliche) Publikation, wie wissenschaftliche Artikel, Berichte, Empfehlungen usw.
- Daten = gemeinsame Sammlung bzw. Nutzung bzw. Austausch von Daten
- Kommunikation = Anwerbung von Citizen Scientists bzw. Projektkommunikation bzw. Öffentlichkeitsarbeit bzw. gemeinsame Social Media-Kanäle
- Veranstaltungen = Durchführung von (gemeinsamen) Veranstaltungen
- Infrastruktur = Nutzung bzw. Entwicklung bzw. Ankauf von Infrastruktur/Technologie
- Beratung = Erfahrungsaustausch bzw. Empfehlungen
- Wirkung = Erzielung (gesellschaftlicher) Wirkung bzw. Impact

Wie genau haben Sie kooperiert?

Bitte geben Sie Ihre Antwort hier ein:

Gründe für Kooperation

Warum haben Sie mit anderen kooperiert? *

Bitte wählen Sie alle zutreffenden Antworten aus:

- Ressourcenmangel (zu wenig Geld, Zeit oder Personal)
- Fehlende Infrastruktur (z.B. Forschungsdatenmanagement, Plattformen, Technologie)
- Gemeinsames Ziel
- Von Fördergebern gefordert
- Fehlendes Know-how/Expertise
- Erzielen von (gesellschaftlicher) Wirkung
- Nutzung von Netzwerken (z.B. erreichen von mehr Personen oder bestimmter Personengruppe)

- Sonstiges:

Warum würden Sie mit anderen kooperieren?*

Bitte wählen Sie alle zutreffenden Antworten aus:

- Ressourcenmangel (zu wenig Geld, Zeit oder Personal)
- Fehlende Infrastruktur (z.B. Forschungsdatenmanagement, Plattformen, Technologie)
- Gemeinsames Ziel
- Von Fördergebern gefordert
- Fehlendes Know-how/Expertise
- Erzielen von (gesellschaftlicher) Wirkung
- Nutzung von Netzwerken (z.B. erreichen von mehr Personen oder bestimmter Personengruppe)
- Sonstiges:

Welchen Nutzen hatten bzw. haben Sie von der Kooperation?

Bitte geben Sie Ihre Antwort hier ein:

Welche Vorteile könnte Ihnen eine Kooperation bringen? Mit wem würden Sie kooperieren?

Bitte geben Sie Ihre Antwort hier ein:

Herausforderungen bei Kooperation

Gab es Herausforderungen bei der Kooperation? *

Bitte wählen Sie nur eine der folgenden Antworten aus:

- Ja
- Nein

Was waren die Herausforderungen bei der Kooperation? *

Bitte wählen Sie alle zutreffenden Antworten aus:

- Mangelnde oder missverständliche Kommunikation untereinander
- Koordination/Management/Organisation
- Unterschiedliche Arbeitsweise und Abläufe

- Unklare Zuständigkeiten und Verantwortlichkeiten
- Fehlende Unterstützung von Vorgesetzten oder der Institution
- Unterschiedliche Ziele oder Prioritäten
- Unterschiedliche Wissensstände/Expertise
- Sonstiges:

Das möchte ich zu den Herausforderungen der Kooperation noch ergänzen...

Bitte geben Sie Ihre Antwort hier ein:

Barrieren für Kooperation

Warum haben Sie bisher nicht kooperiert? *

Bitte wählen Sie alle zutreffenden Antworten aus:

- Fehlende Kapazitäten/Ressourcen
- Mühsame Koordination/Management/Organisation
- Unterschiedliche Arbeitsweise und Abläufe
- Unklare Zuständigkeiten und Verantwortlichkeiten
- Fehlende Unterstützung von Vorgesetzten oder der Institution
- Unterschiedliche Ziele oder Prioritäten
- Unterschiedliche Wissensstände/Expertise
- Konkurrenzdenken (z.B. Fördermittel, Ideenklau)
- Schlechte Erfahrungen mit vorherigen Kooperationen
- Sonstiges:

Unterstützung und Synergien

Wie könnten Kooperationen im Citizen Science-Netzwerk verstärkt werden? *

Bitte geben Sie Ihre Antwort hier ein:

Ich kann Folgendes in das Citizen Science-Netzwerk einbringen... *

Bitte geben Sie Ihre Antwort hier ein:

Welche neuen Partner sollten als Institutionen ins Citizen Science Network Austria aufgenommen werden?

Bitte geben Sie Ihre Antwort hier ein:

Abschluss

Das möchte ich noch ergänzen...

Bitte geben Sie Ihre Antwort hier ein:

Kooperationen im Citizen Science-Netzwerk

Vielen Dank für die Teilnahme an der Umfrage.

Die Ergebnisse werden zur Weiterentwicklung des Citizen Science-Netzwerks genutzt. Die allgemeinen Ergebnisse werden im Blog auf Österreich forscht und eventuell in einer wissenschaftlichen Publikation veröffentlicht.

Bei inhaltlichen Fragen oder für Feedback zur Umfrage steht Ihnen [Name und E-Mail-Adresse der Wissenschafter*innen] gerne zur Verfügung.

Bei Fragen zum Datenschutz wenden Sie sich gerne an: [Name und E-Mail-Adresse des Datenschutzbeauftragten]

Wir freuen uns darauf, mit eurer Hilfe das Citizen Science-Netzwerk weiterentwickeln zu können.

Das Team von Österreich forscht

Vielen Dank für die Beantwortung des Fragebogens.

2.4. Full questionnaire in English (for information purposes only)

This is the English translation of the questionnaire on cooperation between citizen science projects of the Citizen Science Network Austria: This is not a validated translation. This English questionnaire was not used in the study. It is for information purposes only.

Cooperation in the Citizen Science network

As a citizen science community, we are interested in the further development of the Citizen Science Network Austria. An essential part of the network is the cooperation among each other and the synergies that can arise between initiatives and projects. With this survey, we would therefore like to assess the current status of cooperation and synergies between initiatives and projects in order to develop and offer further support options.

It takes **about 10 minutes** to complete the survey.
We look forward to receiving your answers **by 29 February 2024**.

The results of the survey will be published as a blog post on *Österreich forscht* and an academic publication is also planned. In both cases, no conclusions can be drawn about individual projects. Only general statements are made, such as "Natural science projects on *Österreich forscht* cooperate primarily in the area XY".

If you have any queries, please contact: [researcher’s name and e-mail address]

The *Österreich forscht* team

General information

What is the name of your ongoing or completed citizen science project?

At which organization is, or was, the citizen science project implemented?

Levels and forms of cooperation to date

Have you already cooperated with other citizen science projects in the Citizen Science Network Austria?

(By "cooperation" we mean collaboration on a specific topic or in a project, the organization of joint events, etc.) *

Please select only one of the following answers:

- Yes
- No

Please select all citizen science projects with which you have already cooperated at the following levels.

- Project = implementation of a joint project
- Application = submission of a (joint) funding or project application
- Training = offering joint training courses or training sessions
- Publication = Joint (scientific) publication, such as scientific articles, reports, recommendations, etc.
- Data = joint collection, use or exchange of data
- Communication = recruitment of citizen scientists or project communication or public relations work or joint social media channels
- Events = organization of (joint) events
- Infrastructure = use or development or purchase of infrastructure/technology
- Advice = exchange of experience or recommendations
- Impact = achieving (social) effect or impact

Please specify how you cooperated in detail?

Please enter your answer here:

Reasons for cooperation

Why did you cooperate with others?

Please select all applicable answers:

- Lack of resources (too little money, time or personnel)
- Lack of infrastructure (e.g. research data management, platforms, technology)
- Common goal
- Requested by funding bodies
- Lack of know-how/expertise
- Achieving (societal) impact
- Use of networks (e.g. reaching more people or a specific group of people)
- Other:

Why would you cooperate with others?

Please select all applicable answers:

- Lack of resources (too little money, time or personnel)
- Lack of infrastructure (e.g. research data management, platforms, technology)
- Common goal
- Requested by funding bodies
- Lack of know-how/expertise
- Achieving (societal) impact
- Use of networks (e.g. reaching more people or a specific group of people)
- Other:

How have you benefited from the cooperation?

Please enter your answer here:

What benefits do you see in a future cooperation? Who would you cooperate with?

Please enter your answer here:

Challenges of cooperation

Were there any challenges in the cooperation?

Please select only one of the following answers:

- Yes
- No

What were the challenges of the cooperation? *

Please select all applicable answers:

- Lack of or misunderstandings in communication with each other
- Coordination/management/organization
- Different working methods and processes
- Unclear competencies and responsibilities
- Lack of support from superiors or the institution
- Different objectives or priorities
- Different levels of knowledge/expertise
- Other:

I would like to add this comment on the challenges of cooperation...

Please enter your answer here:

Barriers to cooperation

Why have you not cooperated so far?

Please select all applicable answers:

- Lack of capacity/resources
- Tedious coordination/management/organization
- Different working methods and processes
- Unclear competencies and responsibilities
- Lack of support from superiors or the institution
- Different objectives or priorities
- Different levels of knowledge/expertise
- Competitive thinking (e.g. funding, theft of ideas)
- Bad experiences with previous collaborations
- Other:

Support and synergies

How could cooperation within the Citizen Science Network be strengthened?

Please enter your answer here:

I can contribute the following to the Citizen Science Network...

Please enter your answer here:

Which new partners should be new institutions in the Citizen Science Network Austria?

Please enter your answer here:

End of questionnaire

If you would like to add anything,...

Please enter your answer here:

Cooperation in the Citizen Science Network

Thank you for taking part in the survey.

The results will be used to further develop the Citizen Science Network Austria. The general results will be published in the blog on *Österreich forscht* and maybe in an academic publication.

If you have any questions about the content or feedback on the survey, please contact [researcher's name and e-mail address].

If you have any questions about data protection, please contact: [e-mail of data protection officer]

We look forward to further developing the Citizen Science Network Austria with your help.

The *Österreich forscht* team

Thank you for answering the questionnaire.

3. Inclusion and exclusion criteria

For the answers received, the following inclusion and exclusion criteria were applied:

- Projects that are not listed on the *Österreich forscht* platform and thus not affiliated with the Citizen Science Network Austria were excluded from the analysis.
- Follow-up projects, i.e. projects that basically have the same topic and same design are not included, unless they are also listed separately on the *Österreich forscht* platform.
- The projects mentioned in the answers to the questionnaire were checked for different spellings or different names to identify projects that are the same (and just changed their name throughout the project) or used the English name instead the German one.
- In the case of projects that were contained twice or three times in the answers, the project coordinators were contacted directly and asked which of the answers should be included in the data analysis.
- In the case where projects were allocated to several different organizations, the project coordinators were contacted to assess the leading organization.
